# Global Activation of CD8^+^ Cytotoxic T Lymphocytes Correlates with an Impairment in Regulatory T Cells in Patients with Generalized Vitiligo

**DOI:** 10.1371/journal.pone.0037513

**Published:** 2012-05-23

**Authors:** Yang Lili, Wei Yi, Yang Ji, Sun Yue, Shi Weimin, Li Ming

**Affiliations:** 1 Departments of dermatology, Zhongshan Hospital, Fudan University, Shanghai, China; 2 Departments of dermatology, Shanghai First People's Hospital, Shanghai Jiaotong University, Shanghai, China; New York University, United States of America

## Abstract

Melanocyte-specific CD8^+^ cytotoxic T lymphocytes (CTLs) play a pivotal role in vitiligo-induced depigmentation. Yet, the mechanisms underlying the high frequency of generalized autoimmune disorders associated with generalized vitiligo (GV) are unknown. We hypothesized that an imbalance between activated CD8^+^ CTLs and regulatory T cells (Tregs) exists in patients with GV . Assessment of the circulating CD8^+^ CTLs and Tregs by flow cytometric analysis revealed an obvious expansion of CD8^+^ CTLs and a concomitant decrease in Treg cells in GV patients. The percentages of skin infiltrating CD8^+^ CTLs and Tregs were evaluated by immunohistochemistry and revealed dramatically increased numbers of both CD8^+^ CTLs and Tregs in the perilesional skin of GV patients. However, peripheral Tregs were impaired in their ability to suppress the proliferation and cytolytic capacity of autologous CD8^+^ T cells, suggesting that a functional failure of Tregs and the hyper-activation of CD8^+^ CTLs may contribute to progressive GV. Our data indicate that reduced numbers and impaired function of natural Tregs fail to control the widespread activation of CD8^+^ CTLs, which leads to the destruction of melanocytes and contributes to the elevated frequency of various associated autoimmune diseases. This knowledge furthers our understanding of the mechanisms of immune tolerance that are impaired in GV patients and may aid in the future development of effective immunotherapy for GV patients.

## Introduction

Vitiligo is an acquired depigmentation disorder characterized by the loss of melanocytes from the epidermis. This condition affects approximately 0.5–1% of the world's population [1]. The exact etiology of vitiligo remains obscure, but autoimmune factors have been strongly implicated in the development of the disease, especially in generalized vitiligo (GV), because approximately 30% of vitiligo patients are affected with at least one additional autoimmune disorder [2].

CD8^+^ T cell-mediated tissue damage has been demonstrated in common organ-specific autoimmune diseases, such as type I diabetes and multiple sclerosis, and a role for CD8^+^ T cells has been postulated in the pathogenesis of GV . Previous studies have largely focused on melanocyte-specific cytotoxic T lymphocytes (CTLs) and identified their pivotal role in inducing melanocyte destruction [3]–[5]. The presence or activation of melanocyte-specific CTLs, however, does not explain why GV patients often present with other generalized autoimmune conditions, such as autoimmune thyroid disease, Addison's disease, systemic lupus erythematosus and pernicious anemia [2]. Several reports have shown that increases in globally activated CD8^+^ CTLs correlate with disease activity in various autoimmune disorders [6], [7]. We therefore hypothesized that the skin depigmentation of GV patients resulted from a global expansion of activated CD8^+^ CTLs that progressively destroyed melanocytes and led to a high frequency of associated generalized autoimmune diseases. Thus far, the immune mechanisms underlying the induction and activation of autoreactive CD8^+^ CTLs and the loss of tolerance to auto-antigens are not clear.

CD4^+^ CD25^+^ CD127^−^ Foxp3^+^ regulatory T cells (Tregs) are important in maintaining self-tolerance and regulating immune responses in both physiological and disease conditions [8]. Accumulating data indicate that a deficiency or dysfunction of Tregs is associated with impaired immune homeostasis and the development of autoimmune diseases. To date, few papers have investigated Treg numbers or function in GV patients. One report revealed a defect in Treg cell homing to the skin, based on the finding of drastically reduced Treg numbers in vitiligo skin without any systemic drop in their abundance or activity [9]. In contrast, a recent report identified increased numbers of Tregs in perilesional skin despite a functional defect of circulating Tregs in progressive vitiligo [10]. Whether the prevalence and/or function of Tregs are truly impaired in GV patients is still controversial. Moreover, studies presenting the reciprocal relationship between CD4^+^ CD25^+^ CD127^−^ Foxp3^+^ Tregs and CD8^+^ CTLs in GV progression are lacking.

To address these issues, 50 GV patients were enrolled in this study. The frequencies of Tregs and CD8^+^ CTLs were analyzed in serum or skin samples of GV patients with progressive or stable disease, respectively. The ability of Tregs to suppress polyclonal CD8^+^ T cell responses was also assessed using cells from GV patients. Our results showed that CD8^+^ CTLs that express interferon-γ (IFN-γ), Granzyme B (GrB) and Perforin exhibited a global expansion, whereas circulating CD4^+^ CD25^+^ CD127^−^ Treg cells were significantly reduced among the peripheral blood mononuclear cells (PBMCs) of GV patients. Depletion of natural Tregs was related to the expansion of CD8^+^ CTLs. Moreover, while both CD8^+^ T and Foxp3^+^ Tregs were increased in the perilesional skin of GV patients, we reasoned that GV patients' Tregs would exhibit a functional failure based on the observation that circulating Tregs failed to effectively suppress CD8^+^ T cell proliferation and/or the release of cytolytic molecules. Our data indicate that the pathophysiology of GV, and its associated increase risk of autoimmune disorders, might be linked to a defect in the homeostatic control of activated CD8^+^ CTLs and/or natural Treg cell subpopulations.

## Results

### Highly enriched circulating CD8^+^ cytotoxic T lymphocytes in patients with GV

**Figure 1:**
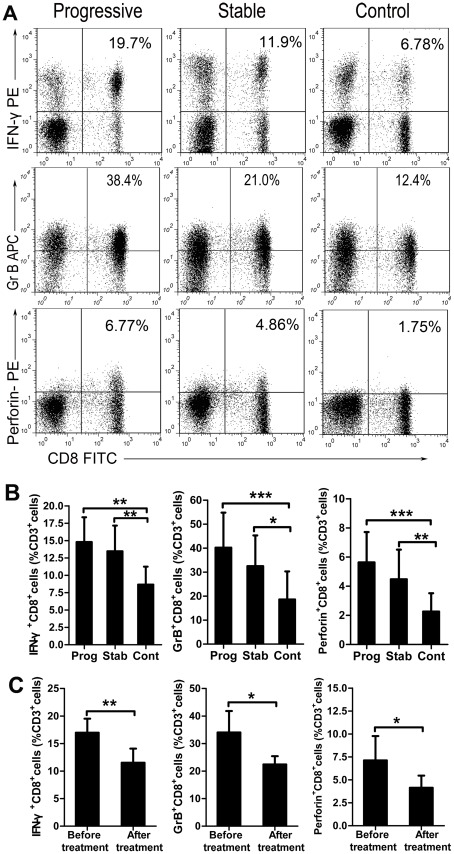
Circulating CD8^+^ CTLs are increased significantly in GV patients. (A) IFN-γ, GrB and Perforin–expressing cells were detected by intracellular staining. Values are the percentage of IFN-γ^+^ CD8^+^, GrB^+^ CD8^+^ or Perforin^+^ CD8^+^ cells among CD3^+^ T cells (Representative cases). (B) Flow cytometric analysis of IFN-γ^+^ CD8^+^, GrB^+^ CD8^+^ and Perforin^+^ CD8^+^ cells within the CD3^+^ T cell populations from patients with progressive GV (n = 38), stable GV (n = 12) and control subjects (n = 20). * = P<0.05, ** = P<0.01, *** = P<0.001. (C) The CD8^+^ CTL population of 6 patients was monitored longitudinally: The percentage of IFN-γ^+^ CD8^+^ T cells, GrB^+^ CD8^+^ T cells and Perforin^+^ CD8^+^ T cells was measured initially during GV progression and again following stabilization after treatment. * = P<0.01, ** = P<0.001.

Effector CD8^+^ CTLs play a key role in the host defense through the production of cytokines, such as IFN-γ, and the effector molecules, GrB and Perforin. CD8^+^ CTLs can also support deleterious effects when they react against self tissues. As the actual functional molecules for killing target cells, IFN-γ, GrB and Perforin can be used as markers for effector CD8^+^ CTLs [11]. In our study, PBMCs obtained from 38 progressive GV patients, 12 stable GV patients, and 20 normal controls were analyzed for the frequency of CD3^+^ CD8^+^ T cells and the expression of IFN-γ, GrB and Perforin by flow cytometry (Figure 1A). There was a significantly higher number of circulating IFN-γ^+^ CD8^+^ T among CD3^+^ T cells in progressive GV (mean ± SD 14.80±3.56%; P<0.01) and stable GV (13.46±3.69%; P<0.01) patients compared to healthy individuals (8.66±2.59%) (Figure 1B, left). The percentage of GrB^+^ CD8^+^ T cells among circulating CD3^+^ T cells was significantly increased in active GV (40.16±14.60%; P<0.001) and inactive GV (32.47±12.77%; P<0.05) patients compared to healthy individuals (18.59±11.73%) (Figure 1B, middle). In addition, we observed that Perforin expression in the CD3^+^ CD8^+^ T cell subset was significantly higher in GV patients (progressive GV, 5.64±2.08%, P<0.001; stable GV, 4.48±2.03%, P<0.01) than in normal controls (2.25±1.26%) (Figure 1B, right). Interestingly, no significant difference in the numbers of circulating IFN-γ^+^ CD8^+^ T cells, GrB^+^ CD8^+^ T cells and Perforin^+^ CD8^+^ T cells in CD3^+^ T cells was observed between patients with progressive GV and stable GV (Figure 1B).

We next questioned whether the percentage of CD8^+^ CTLs varied within the same individual in relation to disease status. To assess this, 6 individuals with progressive GV were tested longitudinally; there was a significant decrease in the percentage of CD8^+^ CTLs after the patient's disease was stabilized by treatment with oral traditional Chinese herbs and topical steroids (Figure 1C).

### Decrease of circulating CD4^+^ CD25^+^ CD127^−^ Treg cells in GV patients

**Figure 2:**
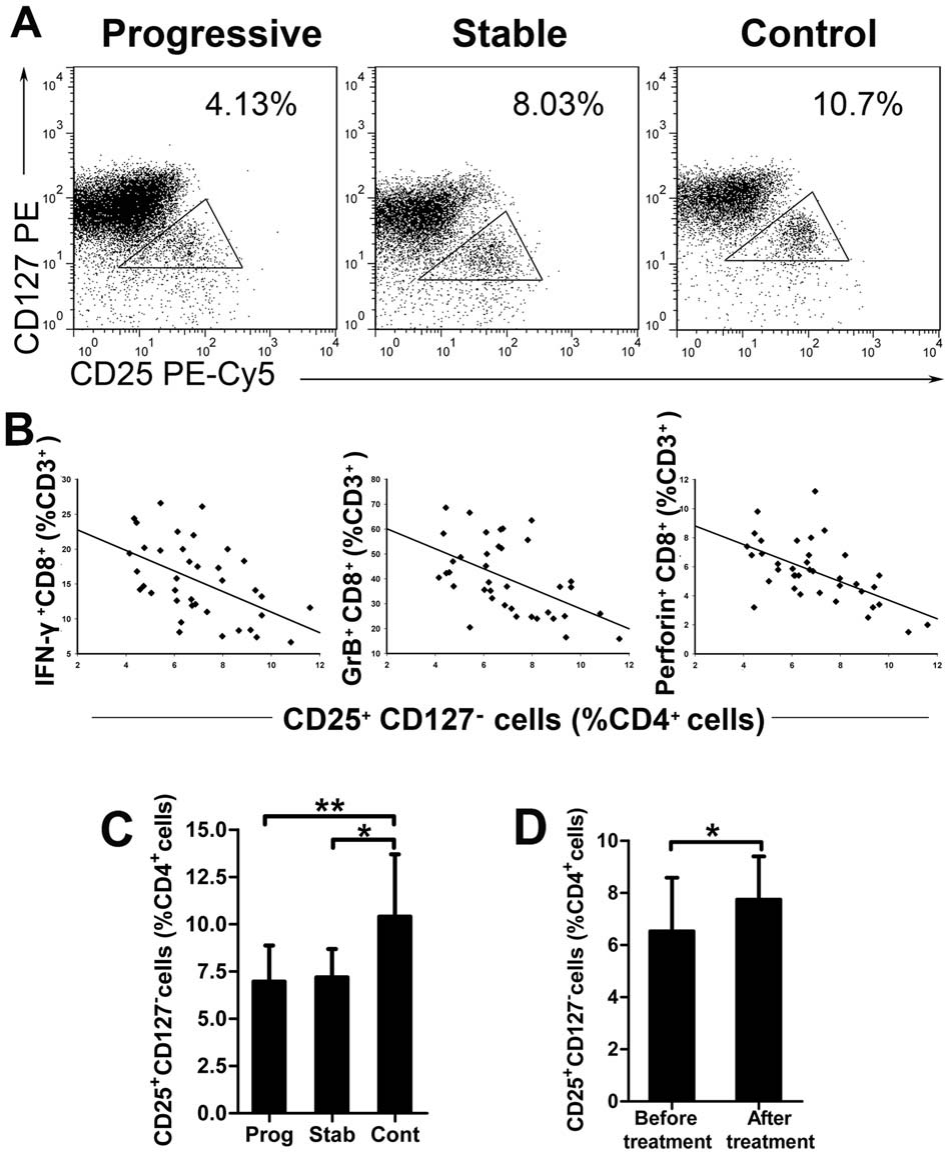
Circulating CD4^+^ CD25^+^ CD127^−^ Tregs are decreased significantly in GV patients. (A) Human PBMCs were labeled with lymphocyte-specific antibodies (CD4, CD25 and CD127). Representative flow data showing the gating strategy and percent of natural Treg cells within the CD4^+^ T cell population are given for one patient from each of the 3 studied groups. (B) A negative correlation was found between the frequency of CD4^+^ natural Treg cells and the expression of IFN-γ (*r* = −0.512, P = 0.001), GrB (*r* = −0.524, P = 0.001) or Perforin (*r* = −0.585, P<0.001) from CD3^+^ CTLs in patients with progressive GV (n = 38). (C) The results of the flow cytometric analysis of natural Treg cells in patients with progressive GV (n = 38), stable GV (n = 12) and control subjects (n = 20) are shown. * = P<0.01, ** = P<0.001. (D) Longitudinal monitoring of natural Treg cells in 6 patients. The percentage of CD4^+^ natural Treg cells was measured twice: once during a vitiligo flare and once following resolution after treatment. * = P<0.05.

Natural Treg cells play a key role in peripheral immune tolerance and prevent the occurrence of autoimmune diseases. In this study, natural Treg cells were quantified by flow cytometric analysis, according to their CD4, CD25 and CD127 surface marker expression [12] (Figure 2A). The percentage of CD4^+^ CD25^+^ CD127^−^ T cells was significantly decreased in the 38 patients with progressive GV (7.0%±1.8%, P<0.001) and the 12 patients with inactive disease (7.2%±1.4%, P<0.01) compared to the 20 healthy controls (10.4%±3.2%) (Figure 2C). However, the numbers of circulating Tregs did not differ significantly between progressive GV and stable patients (P>0.05). We compared the frequency of natural Treg cells among CD4^+^ T cells and the frequency of IFN-γ^+^ CD8^+^, GrB^+^ CD8^+^ or Perforin^+^ CD8^+^ cells among CD3^+^ T cells in progressive GV patients, revealing a strong negative correlation between these two cell populations (Spearman correlation coefficient, *r* = −0.512, P = 0.001, n = 38; *r* = −0.524, P = 0.001, n = 38; *r* = −0.585, P<0.001, n = 38) (Figure 2B).

We next sought to determine whether the percentage of natural Treg cells varied within the same individual in relation to disease status. Six individuals were tested twice, once during active GV and once after their diseases were stabilized with treatment. The percentage of natural Treg cells within these patients increased significantly after treatment (P<0.05) (Figure 2D).

### Increased frequency of CD8^+^ T cells and Tregs in GV skin tissue samples

**Figure 3:**
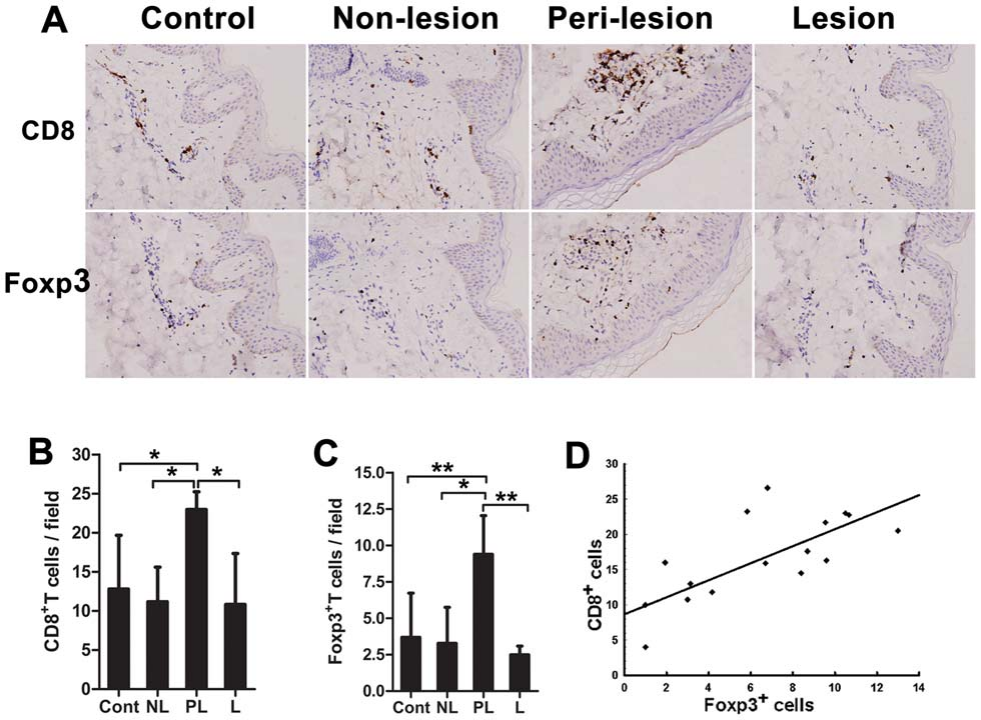
Skin-infiltrating CD8^+^ cells and Foxp3^+^ cells in GV patients. (A) Consecutive sections were used for immunohistochemical detection of T cells expressing CD8 or Foxp3^+^ in skin samples from healthy controls or GV patients (representative fields, 200×). Positive cells appear brown. Both CD8^+^ (B) and Foxp3^+^ (C) T cell numbers were increased significantly in the perilesional (PL) tissues of progressive GV patients (n = 16). In contrast, no significant differences were observed between lesional (L) (n = 2) and non-lesional GV skin (NL) (n = 8), as compared to normal skin from unaffected adults (Cont) (n = 6). * = P<0.05, ** = P<0.01. (D) The number of CD8^+^ and Foxp3^+^ T cells in perilesional GV skin samples exhibited a positive correlation (*r* = 0.706, P<0.001; n = 16). Values in B–C are the mean and SD.

To visualize CD8^+^ T cells and Treg cells in the skin of GV, we performed immunohistochemical staining for CD8 and Foxp3 expression. The number of cells staining positive for these markers were identified in skin tissue samples from normal controls or in non-lesional, perilesional and lesional skin samples from GV patients. Foxp3 is a reliable and specific marker of Tregs because it serves as the dedicated mediator of the genetic program governing Treg cell development and function [13]–[15]. We observed that perilesional skin samples from patients with progressive GV exhibited pathologic changes including a large number of infiltrating CD8^+^ T and Foxp3^+^ cells predominantly adjacent to the epidermal basal layer (Figure 3A).

CD8^+^ T cell counts in each high-power field (400×) were significantly increased in 16 perilesional skin samples from patients with progressive GV (mean ± SD 22.96±2.29), compared to skin from 6 healthy individuals (12.79±6.89, P<0.05), the 2 lesional skin samples from patients with stable disease (10.83±6.53, P<0.05) or 8 non-lesional GV skin samples (11.19±4.4, P<0.05). However, no significant difference was observed between lesional skin and non-lesional skin from GV patients or healthy skin from controls (P>0.05) (Figure 3B). In contrast to previous studies [10], we observed that Foxp3^+^ lymphocyte infiltration was also significantly increased in the 16 perilesional skin samples from patients with progressive GV (9.39±2.66) compared to any of the other sample types: skin samples from 6 healthy individuals (3.69±3.04, P<0.01), lesional skin from patients with stable disease (n = 2; 2.49±0.6, P<0.01) or non-lesional GV skin (n = 8; 3.28±2.47, P<0.05) (Figure 3C). No significant difference was observed between the lesional and non-lesional skin of GV patients or healthy skin from control individuals (P>0.05) (Figure 3C). Importantly, we observed a positive correlation between the number of CD8^+^ T cells and Foxp3^+^ cells in 16 perilesional skin samples from progressive GV patients (Spearman correlation coefficient, *r* = 0.706, P<0.001) (Figure 3D). These results provide evidence that Tregs can be recruited to inflammatory sites from the circulation, and the increase in local CD8^+^ T cells and Foxp3^+^ cells might be involved in the GV disease process.

### Analysis of Treg-mediated suppression of polyclonal CD8^+^ T cell responses

**Figure 4:**
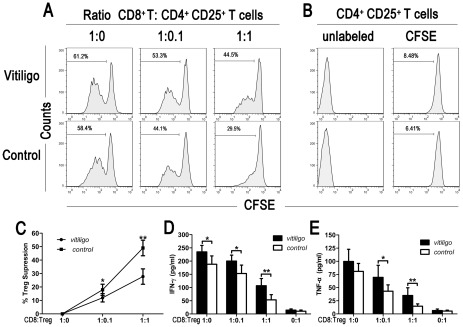
Tregs suppress proliferation and cytokine production of autologous CD8^+^ T cells. (A) Detection of Treg suppression for CD8^+^ T cells. CFSE-labeled CD8^+^ T cells were stimulated in the presence of CD4^+^ CD25^+^ Treg cells at the indicated ratios for 5 days. Cellular division was measured by flow cytometric analysis based on the intensity of CFSE signal. Representative CFSE profiles from a GV patient and a normal control are shown. Values given represent the percentage of CFSE^low^ cells among CFSE^+^ T cells. (B) Treg cells are largely unresponsive to TCR stimulation. Unlabeled and CFSE labeled CD4^+^ CD25^+^ T cells were cultured with anti-CD3/CD28 Abs, under the same stimulatory conditions as above, but without autologous CD8^+^ T cells present. Values given are the percentage of CFSE^low^ cells among CFSE^+^ Treg cells (Representative cases). (C) Dose-dependent suppression of CD8^+^ T cell proliferation by CD4^+^ CD25^+^ T cells. The suppressive capacity of CD4^+^ CD25^+^ Tregs was compared between GV patients (n = 6) and normal control subjects (n = 4). * = P<0.05; ** = P<0.01. The production of IFN-γ (D) and TNF-α (E) was determined. CD8^+^ T cells were cultured with Tregs at various ratios in the presence of anti-CD3/CD28 Ab stimulation for 48 hours. IFN-γ and TNF-α release into the supernatant was determined by an enzyme-linked immunosorbent assay. The data were from 6 progressive GV individuals and 4 healthy controls. * = P<0.05; ** = P<0.01.

Treg/CD8^+^ T cell cocultures were used to evaluate the in vitro suppressive capacity of Tregs toward CD8^+^ T cells using magnetically sorted CD8^+^ T cells and CD4^+^ CD25^+^ Treg cells from either progressive GV patients (n = 6) or normal control subjects (n = 4). T cells were labeled with CFSE and cocultured in the presence of anti-CD3 and anti-CD28 antibodies (anti-CD3/CD28 Abs) at the ratio of either 1∶0.1 or 1∶1 (Figure 4A). By analyzing CFSE intensity by flow cytometric analysis on day 5, we detected a dose-dependent suppression of autologous CD8^+^ T cells by CD4^+^ CD25^+^ Treg cells from both normal controls and GV patients. However, Tregs from GV patients showed a significantly lower suppressive capacity than that of normal controls (mean ± SD 11.97±3.08% vs. 17.92±4.15% at a ratio of 1∶0.1, P<0.05; 27.76±5.71% vs. 49.03±5.80% at a ratio of 1∶1, P<0.01) (Figure 4C). In addition, the CD4^+^ CD25^+^ T cells of normal controls and the majority of GV patients (5/6) were hyporesponsive to polyclonal TCR stimulation when cultured alone, as the majority of Tregs remained CFSE^high^ (Figure 4B). These data indicate that CD4^+^ CD25^+^ Treg cells in progressive GV patients could not effectively suppress CD8^+^ T cell proliferation.

Interestingly, when cocultured with autologous Tregs, CD8^+^ T cells of GV patients had markedly enhanced levels of IFN-γ and TNF-α upon stimulation with anti-CD3/CD28 Abs compared to healthy individuals (Figure 4D and E). These findings suggest that the Tregs of GV patients may not effectively suppress the production of certain cytokines involved in CD8^+^ T cell activation.

## Discussion

A functional CD8^+^ T cell response is an essential component of the adaptive immune response to various cancers and pathogens [16]. Upon engagement with antigen, naive CD8^+^ T cells rapidly expand and differentiate into effector CD8^+^ T cells, which produce cytokines, including IFN-γ, and effector molecules, such as GrB and Perforin, that mediate a local inflammatory response and effect target cell apoptosis [17], [18]. Although effector cytotoxic T lymphocytes (CTLs) play a key role in host defense, they can be detrimental when they react against self tissues. CD8^+^ T cell-mediated tissue damage has been demonstrated in many autoimmune diseases, including vitiligo [19], [20]. Substantial evidence suggests that increased numbers of melanocyte antigen-reactive CD8^+^ T cells contribute to the pathogenesis of GV [3], [21], [22]. However, they fail to explain why generalized autoimmune diseases are present at elevated frequencies among GV patients.

According to a large survey on the epidemiology of vitiligo and associated autoimmune diseases in 2,624 Caucasian probands, approximately 30% of vitiligo patients reported at least one associated autoimmune diseases [2]. The most frequent vitiligo-associated disorder was autoimmune thyroid disease, principally Hashimoto's thyroiditis, occurring in more than 19% of adult Caucasian vitiligo probands. Pernicious anemia, Addison's disease, systemic lupus erythematosus and inflammatory bowel disease all occurred at significantly increased frequencies in vitiligo probands. We previously performed a preliminary survey (unpublished) on associated autoimmune diseases and allergic diseases in 118 Chinese GV patients and observed that 35.59% of GV patients declared at least one associated autoimmune disease or allergic disease. The most frequent vitiligo-associated disease was thyroiditis, occurring in 14.41% of patients. Other autoimmune or allergic phenomena reported in this GV cohort were as follows: urticaria 11.02%, allergic rhinitis 11.02%, asthma 6.78%, eczema 2.54%, systemic lupus erythematosus 0.85% and psoriasis 0.85%. These results confirm previous evidence of an association between GV and other autoimmune diseases [23], [24] and clarify the magnitude of these associations in Caucasian and Chinese patients.

CD8^+^ T lymphocytes play a significant role in autoimmune disorders, as demonstrated in several reports that identify that an increase in the number of activated CD8^+^ T lymphocytes expressing Perforin and GrB correlates with disease activity in patients with systemic lupus erythematosus [6] and multiple sclerosis [7]. These data suggest that widespread activation of CD8^+^ CTLs might be involved in disease flare-ups. We therefore hypothesized that global activation of CD8^+^ CTLs may correlate with GV disease activity and that this activation was responsible for the progress of depigmentation and an increased frequency of associated autoimmune disorders in GV patients. Thus, our studies were designed to delineate the ability of circulating CD8^+^ T cells from GV patients to proliferate and/or produce cytotoxic molecules and cytokines upon stimulation with CD3/CD28 Abs.

In our study, we detected effector CD8^+^ CTLs in the circulation of GV patients, based on their expression of IFN-γ, GrB and Perforin. These specific effector molecules are frequently used in equine research as a surrogate marker for cytotoxic cell activity [25] because studies have shown that their expression correlates well with CD8^+^ T cell cytotoxic activity in PBMC cultures [26]. Herein, we show that patients with progressive GV exhibit an increased proportion of CD8^+^ cells expressing IFN-γ, GrB and Perforin compared to healthy individuals (Figure 1). This demonstrates an increased prevalence of activated CD8^+^ CTLs among PBMCs in GV patients, especially in patients with progressive disease. Furthermore, our data show that an increased proportion of CD8^+^ T cells also accumulate in the perilesional skin of progressive GV patients, especially in areas adjacent to disappearing melanocytes. These data suggest that CD8^+^ CTLs could migrate to sites of inflammation and participate in the destruction of melanocytes, thereby promoting vitiligo-associated depigmentation (Figure 3). Taken together, these data demonstrate that the proportion of CD8^+^ CTLs in both circulating and perilesional skin are enhanced in the setting of progressive GV, suggesting that a global expansion of CD8^+^ CTLs might be involved in the development of GV. In addition, we observed that the prevalence of circulating CD8^+^ CTLs decreased significantly after efficacious treatment with oral Chinese herbs and topical steroids. Thus, the frequency of activated CTLs correlates with disease activity, whereas a normalization of this frequency may precede the clinical stabilization of GV and may be used as a predictive factor of disease outcome.

In the proliferation assay, CD8^+^ T lymphocytes from progressive patients cultured alone showed hyper-activation and produced more IFN-γ and TNF-α upon stimulation with anti-CD3/CD28 Abs, compared with CD8^+^ T cells from control individuals. These data suggest that the cytotoxic function of CD8^+^ T cells may be hyperactive in progressive GV patients (Figure 4). We hypothesized that a defect in immunoregulation resulted in the global activation of effector CD8^+^ CTLs that consequently contributed to elevated frequencies of autoimmune diseases associated with GV. Taken together, our data disclose a new and pivotal role of globally activated CD8^+^ CTLs in GV pathogenesis.

Natural Treg cells play a key role in maintaining peripheral tolerance in vivo through the active suppression of self-reactive T cell activation and expansion [9]. A deficiency or dysfunction of Tregs has been described in several autoimmune diseases, both systemic [27], [28] and organ-specific [29]. Currently, very little is known about the function of Treg cells in GV patients. In this study, we show that GV patients exhibit a decreased prevalence of circulating Tregs (Figure 2) and an increased proportion of perilesion-infiltrating Treg cells (Figure 3). Thus, regulatory T cells are likely recruited to inflamed skin lesions from the peripheral blood, despite a deficiency of circulating Tregs. These data are consistent with current findings demonstrating that circulating Tregs could migrate to sites of inflammation to balance the autoimmune response in progressive GV [10]. Our data, however, conflict with the findings reported by Klarquist et al. [9], who did not notice any deficiency in peripheral Treg cell numbers in vitiligo patients. Rather, they reported a reduced proportion of Tregs in skin samples from vitiligo patients, leading them to hypothesize that circulating Tregs are not recruited to vitiligo skin lesions. This discrepancy may be explained by the small number of patients included in their study. Nonetheless, we observed that the percentage of circulating Treg cells increased significantly after treatment-induced stabilization, suggesting that these Tregs reflected disease activity, and could therefore be a predictive factor for the stabilization of the disease.

Interestingly, the abundance of Tregs in perilesional skin could not prevent CD8^+^ T cell-mediated cytotoxic activity toward melanocytes in progressive GV. Previous studies in some autoimmune disorders have demonstrated that defective regulatory T cell function might result in autoimmune activation [30]. This led us to evaluate the ability of circulating natural Tregs from GV patients to control CD8^+^ T cell proliferation and activation. The suppressive effects of peripheral T regulatory cells in 6 progressive GV cases were significantly reduced, demonstrating an impairment in their ability to inhibit the proliferation and cytokine production of stimulated autologous CD8^+^ T cells. Thus, a functional defect in Tregs might be involved in the pathogenesis of GV disease (Figure 4). For instance, a higher recruitment of functionally deficient Tregs in situ would not be able to regulate the widespread activation of self-reactive CD8^+^ CTLs that mediate the progressive loss or destruction of melanocytes in GV patients and would also explain the elevated frequency of associated generalized autoimmunity. Our findings are consistent with a recent report from Ben Ahmed [10] in which the suppressive effects of peripheral CD4^+^ CD25^+^ T regulatory cells on conventional CD4^+^ CD25^−^ T cell proliferation were analyzed in vitiligo patients. These data revealed a functional defect in Treg cells from 7 of 15 cases. Taken together with our findings, these data indicate the presence of functional defects in Treg cells in patients with progressive GV, which would permit an active immune response against melanocytes and other self-antigens to proceed unhindered, despite the presence and recruitment of circulating peripheral Tregs in sites of diseases activity.

To demonstrate the relationship between the proportion of circulating cytotoxic CD8^+^ T cells and natural Treg cells, we plotted the percentages of peripheral blood IFN-γ^+^ CD8^+^ T cells, GrB^+^ CD8^+^ T cells and Perforin^+^ CD8^+^ T cells against that of Treg cells from progressive GV patient (Figure 2C). Our data showed that these three CD8^+^ CTL populations all correlated negatively with Tregs, suggesting a possible causal association between CD8^+^ CTLs and Tregs in the development of GV. Although concluding whether CD8^+^ CTL hyperactivity is a cause or a consequence of the quantitative and functional defects of Tregs requires further investigation, we hypothesize that deficiencies and dysfunction in the Treg population fail to maintain peripheral tolerance and lead to a copious expansion and activation of self-reactive CD8^+^ CTLs, which destroys melanocytes and leads to a high frequency of generalized autoimmune diseases in GV patients.

Finally, we did not notice any significant difference in the frequency of circulating CD8^+^ CTLs and CD4^+^ CD25^+^ CD127^−^ Treg cells between patients with progressive and stable GV (Figures 1 and 2). Two reasons may account for this observation: (1) The small sample size of stable GV patients we studied may not be representative of this patient population and needs to therefore be confirmed, and (2) the classification of disease activity among our GV patients might be faulty. At present, disease activity is mainly assessed by the medical history information provided by patients themselves. However, the patients' recollection of disease activity might not be accurate. It is conceivable that minor expansions of old lesions or the appearance of new but small lesions are not always noticed by the patient, especially when these changes occur to lesions that are less conspicuous to the patient (e.g., axillae or back). Moreover, patients with extensive vitiligo involving more than 30% of the total body surface area also find it difficult to assess disease activity. Thus, it is possible that some patients with slow progressive disease were mistakenly classified as having stable vitiligo. Thus, further investigation should aim for more precise disease stage-classification standards.

In conclusion, our findings indicate that the imbalance of CD8^+^ CTLs and natural Tregs in frequency and function might be involved in the progression of GV. The deficiency and dysfunction of natural Treg cell subpopulations lead to a global expansion and widespread activation of the CD8^+^ CTL population, which results in the destruction of melanocytes and an elevated frequency of associated autoimmune diseases in GV patients. This knowledge furthers our understanding of the mechanisms that contribute to the autoimmune status of GV patients and provides a new avenue of investigation that may lead to the development of effective immunotherapies for patients with GV.

## Methods

### Patients and controls

Fifty adult patients (18 women and 32 men, mean ± SD age 38±16.7 years) with a diagnosis of GV were enrolled in the study after giving informed consent. The patients were divided into two groups based on whether the existing lesions were spreading and/or new lesions had appeared within the previous 6 months: an affirmative answer to one or both of those questions led to inclusion of the patient in the progressive GV group, whereas patients with no increase in lesion size or number were included the stable GV group. Thirty-eight patients were classified with progressive GV (14 women and 24 men, age 38±16 years), whereas 12 patients were included in the stable GV group (4 women and 8 men, age 38±20 years). A medication-free period was required for both groups, which consisted of at least 1 month for oral drugs, and 2 weeks for topical medications or phototherapy prior to study procedures. As a control, 20 healthy individuals from Fudan University were enrolled in our study. The protocols involving human subjects were approved by the institutional review boards of Zhongshan Hospital of Fudan University. Informed consent was obtained in writing from all subjects before performing the studies.

For the 10 patients in the group of patients with active GV, we administrated oral traditional Chinese herbs and topical steroids twice a day to stop progression and improve lesions. The herbs complex is a mixture extracts of Rx. Angelicae Sinensis, Rx. Astragali, Fructus Tribuli and Caulis Spatholobi, which appears to be effective in halting disease progression and inducing repigmentation in GV. Three months later, 6 patients in 10 cases reported stabilization of their disease, and blood samples from these 6 patients were obtained again for analysis.

Four-millimeter skin biopsies were obtained from 18 patients with GV. Six healthy tissue specimens were obtained from individuals undergoing orthopedic surgery, and four healthy blood samples were obtained from age- and gender-matched volunteers for cell proliferation assays. The patients provided informed consent prior to tissue collection. The study protocol was reviewed and approved by the Zhongshan Hospital research ethics committee.

Available patient information that was relevant to this study is summarized in Table 1 and Table 2 for patients included in the immunohistochemistry data set and the cell proliferation assay, respectively.

**Table 1:** Patient skin biopsy samples for CD8^+^ cells and Foxp3^+^ cells quantification by immunohistochemistry.

Patient gender	Patient age (yr)	Biopsy site	Perilesion/Lesion/Normal skin	Disease duration (yr)	Disease activity	1^st^ degree relatives with vitiligo	Associated disease*
Female	18	Abdomen	Lesion + Normal	16	Stable	No	No
Male	45	Arm	Lesion + Normal	12	Stable	No	No
Male	34	Thorax	Perilesion + Normal	6	Progressive	No	No
Male	59	Abdomen	Perilesion + Normal	20	Progressive	No	Psoriasis
Male	20	Leg	Perilesion + Normal	1	Progressive	No	AR, Asthma
Female	36	Arm	Perilesion + Normal	24	Progressive	No	No
Male	30	Arm	Perilesion + Normal	3	Progressive	No	AR
Female	40	Abdomen	Perilesion + Normal	26	Progressive	Yes	No
Male	40	Back	Perilesion	3	Progressive	No	No
Male	62	Leg	Perilesion	6	Progressive	Yes	No
Female	69	Arm	Perilesion	30	Progressive	Yes	HT
Female	20	Arm	Perilesion	5	Progressive	No	No
Female	43	Abdomen	Perilesion	23	Progressive	No	HT
Male	30	Arm	Perilesion	10	Progressive	No	No
Female	48	Arm	Perilesion	0.5	Progressive	No	SLE
Male	77	Arm	Perilesion	1.5	Progressive	No	AA, AR
Male	46	Abdomen	Perilesion	0.2	Progressive	No	HT
Male	72	Leg	Perilesion	2	Progressive	No	No

* SLE = systemic lupus erythematosus; AA = alopecia areata; AR = allergic rhinitis; HT = Hashimoto's thyroiditis.

**Table 2:** Patient PBMC samples involved in proliferation assay.

Patient gender	Patient age (yr)	Disease duration (yr)	Disease activity	Lesional Area	1^st^ degree relatives with vitiligo	Associated disease*
Female	49	0.5	Progressive	<1%	No	No
Female	45	20	Progressive	8%	Yes	HT
Male	27	16	Progressive	1%	Yes	No
Male	16	1	Progressive	<1%	No	No
Male	48	10	Progressive	10%	No	HT
Male	18	5	Progressive	8%	Yes	AR, Asthma

* HT = Hashimoto's thyroiditis. AR = allergic rhinitis.

### Flow cytometry

All blood samples were processed on the day of collection. Peripheral blood mononuclear cells (PBMCs) were purified on Ficoll gradients (GE Healthcare, Björkgatan, Sweden). For the detection of natural Treg cells, PBMCs were stained with fluorescein isothiocyanate (FITC)-conjugated anti-CD4, phycoerythrin-Cy5 (PE-Cy5)-conjugated anti-CD25, and PE-conjugated anti-CD127 (eBiosciences, San Diego, CA), according to the manufacturer's protocol. After staining, cells were washed twice in fluorescence-activated cell sorting solution (phosphate buffered saline [PBS] with 0.5% bovine serum albumin [BSA] and 0.02% sodium azide), fixed in PBS containing 1% paraformaldehyde, and collected the same day on a FACSCalibur system (BD Biosciences, San Jose, CA) followed by analysis using FlowJo software (Tree Star, San Carlos, CA). CD25^+^ CD127^−^ natural Treg cells were identified within gated CD4^+^ T cells.

### Intracellular cytokine staining

For the detection of IFN-γ, PBMCs were incubated for 4–5 hours in RPMI 1640 (containing 10% fetal bovine serum [FBS]) with 50 ng/ml phorbol myristate acetate (PMA) and 750 ng/ml ionomycin in the presence of 1.7 µg/ml monensin (Enzo Biochem, Farmingdale, New York) in a tissue culture incubator at 37°C. No incubation was performed for the detection of GrB and Perforin molecules. For intracellular staining of IFN-γ, GrB and Perforin molecules, PBMCs were first stained with PerCP-conjugated anti-CD3 and FITC-conjugated anti-CD8 antibodies and then fixed and permeabilized using FIX & PERM (Invitrogen, Carlsbad, CA), according to the manufacturer's instructions. Permeabilized cells were then stained with either PE-conjugated anti-IFN-γ or APC-conjugated anti-GrB and PE-conjugated anti-Perforin (eBioscience, San Diego, CA) for 20 minutes. Stained cells were then fixed in 1% paraformaldehyde, and 4-color flow cytometric analyses were performed using FACSCalibur and FlowJo software. For the detection of CD8^+^ cytotoxic T cells, IFN-γ^+^ CD8^+^, GrB^+^ CD8^+^ or Perforin^+^ CD8^+^ T cells were analyzed among cells first included in a CD3^+^ gate.

### Immunohistochemistry

Tissues from 18 GV patients were processed and embedded in paraffin using routine methods. Tissue blocks were serially sectioned to obtain consecutive sections. Immunohistochemistry for CD8^+^ and Foxp3^+^ T cells was performed using the Novolink Polymer Detection System (Novocastra, Newcastle-upon-Tyne, UK) according to the manufacturer's instructions. Briefly, paraffin-embedded sections were first deparaffinized and then hydrated. After microwave antigen retrieval, endogenous peroxidase activity was blocked by the incubation of slides in 0.3% H_2_O_2_, whereas nonspecific binding sites were blocked with Protein Block (Novocastra). Primary monoclonal antibodies directed against CD8 (Dako, Copenhagen, Denmark) and Foxp3 (Abcam, Cambridge, UK) were used for CD8 and Foxp3 staining, respectively. Following serial incubations with primary antibodies, Primary Block, and secondary antibody treatment (Novolink polymer; Novocastra), the sections were developed in a diaminobenzidine solution and counterstained with hematoxylin. Sections incubated with PBS only served as negative controls.

For quantification of CD8^+^ and Foxp3^+^ cells, skin sections were examined microscopically at high power (400×). Five high-power fields of each sample were selected for digital photographs. These same five areas were counted manually by two independent observers for the number of CD8^+^ or Foxp3^+^ cells in each field.

### Cell isolation

All blood samples were processed on the day of collection. PBMCs were purified by Ficoll-Hypaque density gradient centrifugation from heparinized blood. CD4^+^ CD25^+^ Tregs were isolated from PBMCs by CD4-negative selection followed by CD25-positive selection, using the CD4^+^ CD25^+^ T-cell isolation kit (Miltenyi Biotech, Teterow, Germany), with MidiMACS and MiniMACS separator units (both from Miltenyi Biotech), according to the manufacturer's instructions. A CD8^+^ T-cell isolation kit (Miltenyi Biotech, Teterow, Germany) was used to isolate CD8^+^ cells by CD8-negative selection. The purities of CD4^+^ CD25^+^ Tregs and CD8^+^ T cells were 90% or greater and 98%, respectively.

### CD8^+^ T cell proliferation assay and cytokine detection

Carboxyfluorescein diacetate succinimidyl ester (CFSE) (Invitrogen Corporation, Carlsbad, CA) was used to detect the proliferation of CD8^+^ T cells according to manufacturer's instruction. Briefly, freshly prepared CFSE stock solution was added to CD8^+^ or CD4^+^CD25^+^ T cell suspensions at a final concentration of 4 µM and then incubated for 10 minutes at 37°C. Cells were washed and resuspended in 5 volumes of ice-cold RPMI 1640 culture medium for 5 minutes on ice to stabilize the CFSE staining. After two washes, CFSE-labeled cells were resuspended and cultured with CD4^+^ CD25^+^ Tregs at various ratios. In a U-bottom 96-well plate coated with 5 µg/ml anti-CD3 Abs (OKT3) (eBioscience, San Diego, CA), CFSE-labeled cells were added at 5×10^4^ cells per well in RPMI 1640 (containing 10% FBS) with 2 µg/ml soluble anti-CD28 Abs (CD28.2) and incubated at 37°C and 5% CO_2_. On day 3 of culture, the culture medium was replaced with fresh RPMI 1640 (containing 10% FBS) with 2 µg/ml soluble anti-CD3 and anti-CD28 Abs. Cells were harvested on day 5, and the intensity of CFSE staining of CD8^+^ T cells or CD4^+^ CD25^+^ Treg populations was determined using a FACSCalibur system (BD Biosciences, San Jose, CA) and FlowJo software (Tree Star, San Carlos, CA). The suppressive capacity of Tregs toward CD8^+^ T cells in coculture (CD8^+^ T cell to Treg ratio of 1∶0.1 or 1∶1) was defined as relative inhibition based on the percentage of CFSE^low^ cells used as a measure of proliferation: [100×(1-% CFSE^low^ CD8^+^ T cells in coculture/% CFSE^low^ CD8^+^ T cells alone)].

For IFN-γ and tumor necrosis factor-α (TNF-α) detection, the plate was cultured for 48 hours. Cytokine release into the supernatant was measured using the corresponding enzyme-linked immunosorbent assay kits (Bender, Vienna, Austria), according to the manufacturer's instructions.

### Statistical analysis

Data analysis was performed with SPSS version 12.0 for Windows software (SPSS Inc., Chicago, IL). Quantitative data were expressed as the mean ± SD. Statistical significance was determined by an analysis of variance followed by the Bonferroni post hoc test for comparisons of multiple means or Student's t-test. Comparisons of the rates of circulating natural Treg cells, IFN-γ^+^ CD8^+^, GrB^+^ CD8^+^ and Perforin^+^ CD8^+^ cells during the evolution of disease were made by paired t-tests. Correlations were determined by Spearman's ranking. A P value of less than .05 was considered significant.

## References

[1] Taieb A, Picardo M, Members V (2007). The definition and assessment of vitiligo: a consensus report of the Vitiligo European Task Force.. Pigment Cell Research.

[2] Alkhateeb A, Fain PR, Thody A, Bennett DC, Spritz RA (2003). Epidemiology of vitiligo and associated autoimmune diseases in Caucasian probands and their families.. Pigment Cell Res.

[3] Ogg GS, Dunbar PR, Romero P, Chen JL, Cerundolo V (1998). High frequency of skin-homing melanocyte-specific cytotoxic T lymphocytes in autoimmune vitiligo.. Journal of Experimental Medicine.

[4] Palermo B, Campanelli R, Garbelli S, Mantovani S, Lantelme E (2001). Specific cytotoxic T lymphocyte responses against Melan-A/MART1, tyrosinase and Gp100 in vitiligo by the use of major histocompatibility complex/peptide tetramers: the role of cellular immunity in the etiopathogenesis of vitiligo.. Journal of Investigative Dermatology.

[5] Lang KS, Caroli CC, Muhm A, Wernet D, Moris A (2001). HLA-A2 restricted, melanocyte-specific CD8(+) T lymphocytes detected in vitiligo patients are related to disease activity and are predominantly directed against MelanA/MART1.. Journal of Investigative Dermatology.

[6] Blanco P, Pitard V, Viallard JF, Taupin JL, Pellegrin JL (2005). Increase in activated CD8+T lymphocytes expressing perforin and granzyme B correlates with disease activity in patients with systemic lupus erythematosus.. Arthritis and Rheumatism.

[7] Giovanni F, Domenico P, Alessandro M, Raffaele I, Viviana N (2011). Circulating CD8(+)CD56(−)perforin(+) T cells are increased in multiple sclerosis patients.. Journal of Neuroimmunology.

[8] Sakaguchi S (2004). Naturally arising CD4(+) regulatory T cells for immunologic self-tolerance and negative control of immune responses.. Annual Review of Immunology.

[9] Klarquist J, Denman CJ, Hernandez C, Wainwright DA, Strickland FM (2010). Reduced skin homing by functional Treg in vitiligo.. Pigment Cell Melanoma Res. England.

[10] Ben Ahmed M, Zaraa I, Rekik R, Elbeldi-Ferchiou A, Kourda N (2011). Functional defects of peripheral regulatory T lymphocytes in patients with progressive vitiligo.. Pigment Cell Melanoma Res.

[11] Wolint P, Betts MR, Koup RA, Oxenius A (2004). Immediate cytotoxicity but not degranulation distinguishes effector and memory subsets of CD8(+) T cells.. Journal of Experimental Medicine.

[12] Liu W, Putnam AL, Xu-Yu Z, Szot GL, Lee MR (2006). CD127 expression inversely correlates with FoxP3 and suppressive function of human CD4+ T reg cells.. J Exp Med.

[13] Fontenot JD, Gavin MA, Rudensky AY (2003). Foxp3 programs the development and function of CD4(+)CD25(+) regulatory T cells.. Nature Immunology.

[14] Hori S, Nomura T, Sakaguchi S (2003). Control of regulatory T cell development by the transcription factor Foxp3.. Science.

[15] Yagi H, Nomura T, Nakamura K, Yamazaki S, Kitawaki T (2004). Crucial role of FOXP3 in the development and function of human CD25(+)CD4(+) regulatory T cells.. International Immunology.

[16] Kaech SM, Wherry EJ, Ahmed R (2002). Effector and memory T-cell differentiation: Implications for vaccine development.. Nature Reviews Immunology.

[17] Trapani JA, Smyth MJ (2002). Functional significance of the perforin/granzyme cell death pathway.. Nature Reviews Immunology.

[18] Shankaran V, Ikeda H, Bruce AT, White JM, Swanson PE (2001). IFN gamma and lymphocytes prevent primary tumour development and shape tumour immunogenicity.. Nature.

[19] Walter U, Santamaria P (2005). CD8(+) T cells in autoimmunity.. Current Opinion in Immunology.

[20] Liblau RS, Wong FS, Mars LT, Santamaria P (2002). Autoreactive CD8 T cells in organ-specific autoimmunity: Emerging targets for therapeutic intervention.. Immunity.

[21] Oyarbide-Valencia K, van den Boorn JG, Denman CJ, Li M, Carlson JM (2006). Therapeutic implications of autoimmune vitiligo T cells.. Autoimmunity Reviews.

[22] Wankowicz-Kalinska A, van den Wijngaard R, Tigges BJ, Westerhof W, Ogg GS (2003). Immunopolarization of CD4(+) and CD8(+) T cells to type-1-like is associated with melanocyte loss in human vitiligo.. Laboratory Investigation.

[23] Cunliffe WJ, Hall R, Newell DJ, Stevenson CJ (1968). VITILIGO THYROID DISEASE AND AUTOIMMUNITY.. British Journal of Dermatology.

[24] Schallreuter KU, Lemke R, Brandt O, Schwartz R, Westhofen M (1994). VITILIGO AND OTHER DISEASES - COEXISTENCE OR TRUE ASSOCIATION - HAMBURG STUDY ON 321 PATIENTS.. Dermatology.

[25] Breathnach CC, Soboll G, Suresh M, Lunn DP (2005). Equine herpesvirus-1 infection induces IFN-gamma production by equine T lymphocyte subsets.. Veterinary Immunology and Immunopathology.

[26] Malyguine A, Shafer-Weaver K, Derby E, Burkett M, Sayers T (2003). Granzyme B and IFN-gamma production by effector cells in cell-mediated cytotoxicity.. European Cytokine Network.

[27] Antiga E, Kretz C, Klembt R, Massi D, Ruland V (2010). Characterization of regulatory T cells in patients with dermatomyositis.. Journal of Autoimmunity.

[28] Miyara M, Amoura Z, Parizot C, Badoual C, Dorgham K (2005). Global natural regulatory T cell depletion in active systemic lupus erythematosus.. Journal of Immunology.

[29] Chi LJ, Wang HB, Wang WZ (2008). Impairment of circulating CD4(+)CD25(+) regulatory T cells in patients with chronic inflammatory demyelinating polyradiculoneuropathy.. Journal of the Peripheral Nervous System.

[30] Westerhof W, d'Ischia M (2007). Vitiligo puzzle: the pieces fall in place.. Pigment Cell Research.

